# Case report: Incidence and prognostic value of brain MRI lesions and elevated cerebrospinal fluid protein in children with Guillain-Barré syndrome

**DOI:** 10.3389/fneur.2022.885897

**Published:** 2022-10-21

**Authors:** Francesco Pizzo, Alessandra Di Nora, Alessia Di Mari, Giuseppe Costanza, Elisabetta Testa, Marianna Strazzieri, Filippo Greco, Tiziana Timpanaro, Antonio Basile, Giuseppe Belfiore, Andrea Giugno, Roberta Rocca, Martino Ruggieri, Agata Fiumara, Piero Pavone

**Affiliations:** ^1^Postgraduate Training Program in Pediatrics, Department of Clinical and Experimental Medicine, University of Catania, Catania, Italy; ^2^Radiology Unit, Department of Medical Surgical Sciences and Advanced Technologies “GF Ingrassia”-University Hospital “Policlinico-Vittorio Emanuele”, University of Catania, Catania, Italy; ^3^Department of Clinical and Experimental Medicine, Section of Pediatrics and Child Neuropsychiatry, University of Catania, Catania, Italy

**Keywords:** Guillain–Barré syndrome, CSF, childhood, MRI, clinical outcome, neurology-clinical

## Abstract

**Background:**

Guillain-Barrè syndrome (GBS) is an acute immune-mediated disorder affecting peripheral nerves and nerve roots with a variable clinical course and outcome. Epidemiologic analyses have revealed that the incidence of the syndrome increases linearly among the age. The clinical diagnosis of GBS is based on the family history, physical and neurological examination, electrodiagnostic exams, and cerebrospinal fluid analysis with the classical presence of albumin-cytologic dissociation. Prognosis is associated with the severity of clinical signs and the type of peripheral nerves involved.

**Methods:**

This study aims to clarify which clinical features can be used for prognostic purposes. We evaluated the correlation between (1) brain MRI lesions and grade of disability; (2) brain MRI lesions and elevated cerebrospinal fluid (CSF) protein; and (3) increased levels of CSF protein and grade of disability. Statistical analysis extracted from these data indicated a good correlation to be a prognostic indicator in children affected by GBS. We found little evidence regarding laboratory tests, imaging, and prognosis. We enrolled 12 continuous patients who met the Brighton criteria for GBS in this retrospective study. Each patient was clinically evaluated at the time of disease onset to assess the GBS disability score and after 2 weeks.

**Results:**

We estimated Pearson's correlation index to evaluate the possible correlation between MRI and disability and CSF protein levels and disability. The correlation coefficient was 0.92 and 0.85, respectively. In addition, we developed a graph to see the trend of the disability values, proteins in the CSF, and damage assessed with MRI in the 12 patients. It seems that these parameters have a parallel trend and a good correlation in each patient. Finally, we calculated the correlation between MRI and CSF protein values, with an *r*-value of 0.87. The values suggest a correlation among the MRI score, CSF protein, and prognosis.

**Conclusion:**

The MRI and CSF laboratory parameters can be important tools for the clinician not only for diagnosis but also to evaluate the possible worsening of general conditions or the need to prepare measures to support life parameters. Patients who need ventilatory support could be established early from patients who have less severe GBS and can begin rehabilitation earlier. We suggest MRI should be performed routinely in children with GBS to be able to estimate the evolution of the clinical condition.

## Introduction

Guillain-Barrè syndrome (GBS) is an immune-mediated rapidly developed polyneuropathy whose etiology and pathogenesis are not yet entirely comprehended. Guillain–Barré syndrome (GBS) manifests clinically as acute flaccid paralysis, marked by the symmetrical weakness of the limbs, and hyporeflexia or areflexia, which arrives full harshness within 4 weeks (1).

Sensory symptoms, such as paraesthesia or insensibility, begin distally and have a symmetrical extension.

Among pediatric patients in the zenith phase of the syndrome, 75% are unable to walk unsupported, 30% are quadriplegic, 35–50% show cranial nerves involvement, and 15–20% have respiratory failure and/or autonomic dysfunctions (2, 3). Mortality, or severe disability due to GBS, occurs in ~20% of patients (4).

Epidemiological analyses have documented that the incidence of GBS increases with age. In children from 0 to 9 years, it occurs with an incidence of 0.62 cases per 100,000 person-year (py) (5, 6).

Diagnosis of GBS is based on the patient's family history, physical and neurological assessment with sensorial and motor disturbances, electrodiagnostic exams, and cerebrospinal fluid (CSF) which reveals the classical pattern of albumin-cytologic dissociation.

The prognosis of the GBS is linked to the severity of clinical signs and unclear to the presence of autonomic nerve dysfunction (7). Predictors of poor outcomes are defined as a GBS Disability Scale score ≥3 after either 2 weeks or 6 months (8).

We evaluated the correlation among CSF analysis, imaging, and the severity of the GBS in the acute phase to predict a short-term outcome.

## Aim of the study

We aimed to evaluate CSF analysis and imaging to determine the severity of the GBS in the acute phase and to predict a short-term outcome.

- We evaluated the possible correlation among MRI radiological classification, the values of proteins in the CSF, and the prognosis of children with GBS.
- The correlation between the increasing values of the radiological classification of the clinic with the GBS disability score and the prognosis was evaluated.
- In addition, we evaluated the correlation between increasing CSF protein levels and symptomatology and prognosis.

## Materials and methods

A systematic revision of the current literature was conducted using Cochrane, EMBASE, and MEDLINE. In addition to official websites of highly qualified journals which were expected to publish studies related to this topic, for example, the New England Journal of Medicine, The Lancet, PLOS Medicine, Neurology, and Pediatrics were also searched for relevant studies. The words used to search were: “Child; Guillain-Barrè; Neurology; Prognosis; Disability; Laboratory; MRI; cerebrospinal fluid.” We found little evidence regarding laboratory tests, imaging, and prognosis. Following, we cite the most recent evidence regarding prognosis and imaging and prognosis and CSF.

Althubaiti et al. in a recent publication on the prognosis value of MRI in children with GBS advise that brain and spinal MRI is a recommended supportive test but a predictive value for clinical and therapeutic outcomes in the short or long term has not yet been proved (9).

Regarding the proteins in the CSF, we want to mention the most recent evidence proposed by Kasser et al. In their letter to the editor, the authors affirmed CSF protein levels seemed to be potentially associated with the need for mechanical ventilation (10).

### GBS disability score

The Guillain-Barré syndrome (GBS) disability score is a widely accepted scoring system to evaluate the functional level of patients with GBS. It was originally described in Hughes et al. (11) and since then, diverse variations have emerged in the literature.

The patient's level of disability is reported operating on a scale from 0 to 6: Grade 0 is assigned to the asymptomatic patient; Grade 1 is associated with movement reduction but capable of performing manual work; Grade 2 is assigned to those who walk but are unable to perform work with their hands; Grade 3 concerns patients who need support for walking; Grade 4 is assigned to patients who are bedridden. The last 2 degrees of the disease in which there is respiratory failure or death (Table 1). The GBS disability score is not only a clinical parameter but also provides a prognostic value. Revealed disability at the onset and the acute phase of the disease were signs of poor long-term prognosis.

**Table 1:** Levels of GBS disability score.

**Score**	**Description**
0	Healthy state
1	Minor symptoms and capable of running
2	Able to walk 10 m or more without assistance but unable to run
3	Able to walk 10 m across an open space with help
4	Bedridden or chairbound
5	Requiring assisted ventilation for at least part of the day
6	Dead

The Erasmus GBS and van Koningsveld et al. (8–12) evaluated the association between clinical severity at onset and short-term prognosis. They introduced three variables that were predictive of poor outcome at 6 months, and inability to walk independently: age, preceding diarrhea, and GBS disability score at 2 weeks after entry.

### Brain MRI lesions

Magnetic resonance (MR) imaging also contributes to the diagnosis of GBS by demonstrating anterior and posterior intrathecal spinal nerve roots and cauda equine. Gorson et al. (13) showed a correlation between the severity of enhancement of the nerve roots and the severity of the clinical grade. Yikilmaz et al. (14) described the MR features in children with GBS and introduced a radiological classification based on the patterns of contrast enhancement, as reported in Table 2.

**Table 2:** MRI imaging contrast enhancement pattern.

I	No enhancement
II	Anterior roots>posterior roots
III	Anterior roots=posterior roots
IV	Only anterior roots enhance

**Figure 1:**
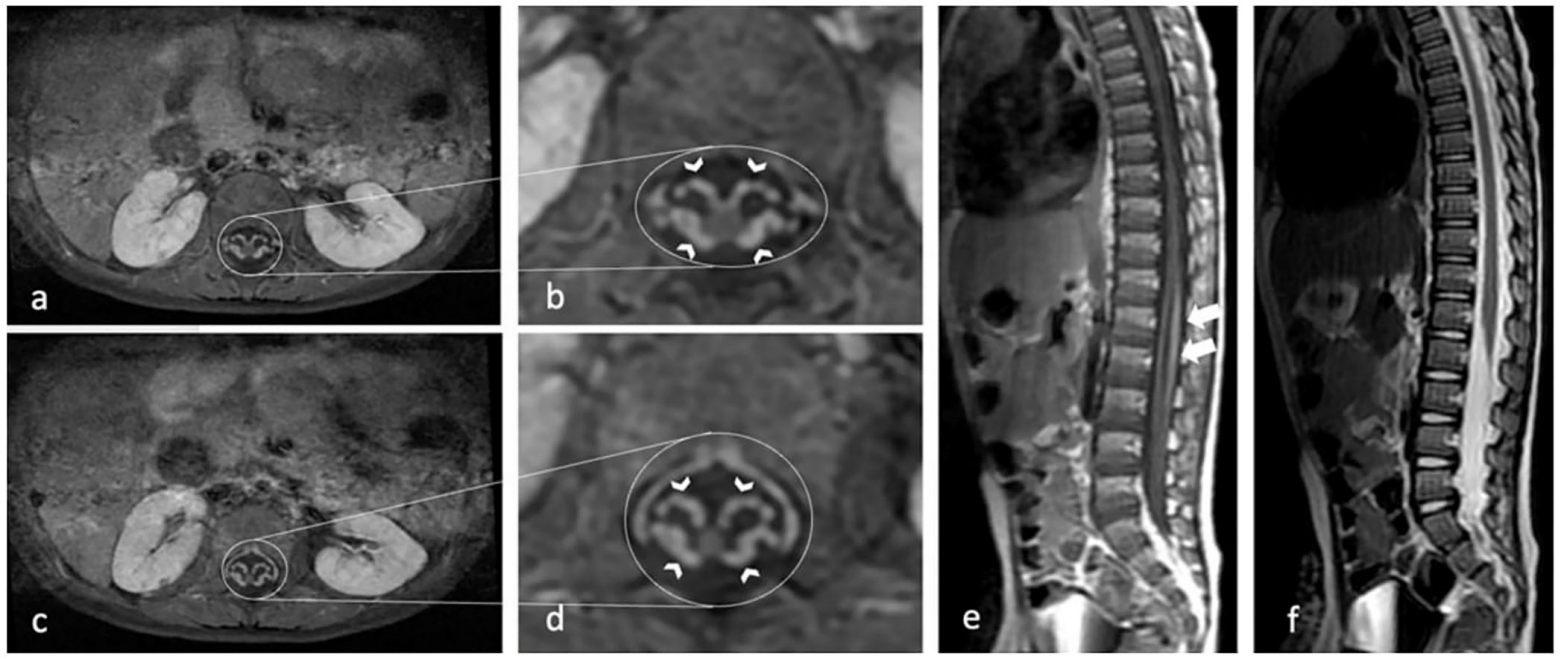
Contrast-enhanced axial T1-weighted MR image shows marked enhancement of the anterior and posterior nerve roots (arrow heads) in the conus medullaris and cauda equina **(a–d)**. Sagittal T1-weighted MR image **(e)** show mild thickening and moderate contrast enhancement of nerve root in the conus medullaris and cauda equina (white arrows). Sagittal T2-weighted MR image **(f)** show mild thickening of cauda equina.

Figure 1 shows one of our patients' axials with a marked enhancement of the anterior and posterior nerve roots and a GBS disability score of 4.

### Elevated CSF protein

We evaluated the chemical-physical characteristics of the CSF in each child, examining appearance, color, number of cells, glucose, proteins, presence of immunoglobulins, and albumin. We examined all these parameters one by one for their correlation with the GBS disability score and therefore with the prognosis.

In this study, the levels of CSF proteins were evaluated according to the increases of 1, 2, and 3 times the basal cut-off value of 45 mg/dl.

### Enrolled patients

The manuscript attempts to evaluate whether a clinical score (the grade of disability of patients), an imaging score (spinal MRI enhancement), and a grade of albumin-cytologic dissociation in CSF are linearly correlated to give a prognostic value of the disease.

**Figure 2:**
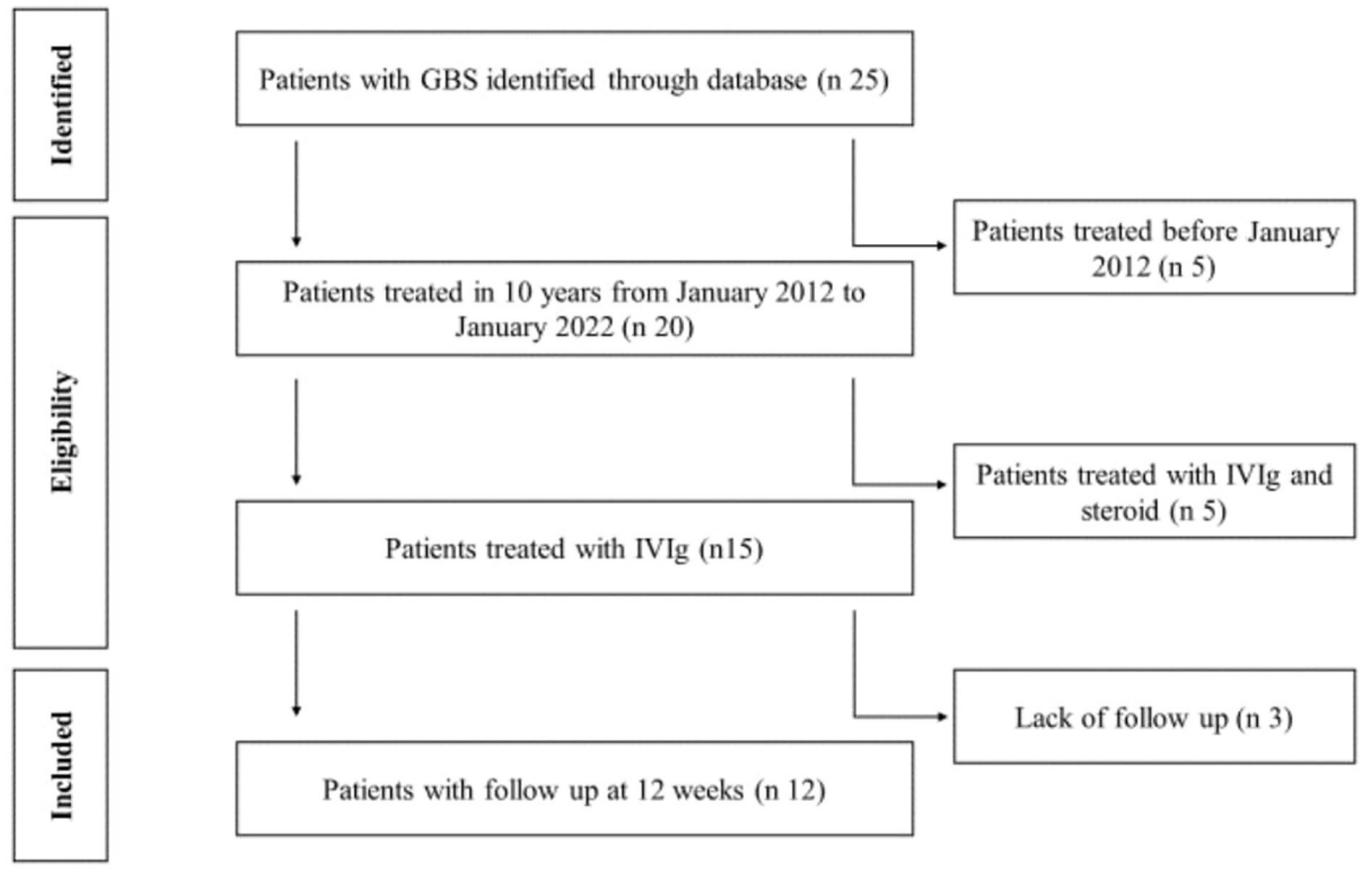
Patients recruitment.

We retrospectively enrolled 12 continuous children affected by GBS and diagnosed at the Clinical Pediatrics Department for 10 years from January 2012 to January 2022 (Figure 2). The children were from 1 year to 14 years old, with a mean age of 6.8 years. The gender ratio was 7 boys to 5 girls. Furthermore, all cases examined met the Brighton criteria validated for children (15). Table 1 reports the grade of disability and other clinical features associated with GBS signs observed in the children. We evaluated the following characteristics of the population included in this study:

- Clinical: each patient was clinically evaluated at the time of disease onset assessing the GBS disability score and after 2 weeks. In addition, all the children treated in our center were placed in instrumental clinical follow-up by carrying out checks at 2 weeks to 1 month, 3 months to 6 months, and 1 year from the onset of the disease. In this study, we collected clinical data at onset and 2 weeks after onset (Table 3).
- Imaging: all patients enrolled in this study underwent MR of the brain and spinal cord. Images were evaluated and classified by the pediatric neuroradiology team and assessed by Yakilmaz radiological classification (14).
- Laboratory: lumbar puncture (LP) was performed on average 3 days after symptom onset (Table 4), and all enrolled patients had CSF analyzed.

**Table 3:** The grade of disability and other clinical features associated to GBS.

**Age (Years)**	**M/F**	**GBS disability score**	**Functional deficit**	**Respiratory deficit**	**Cranial nerves**	**Autonomic system**
14	M	4	Bedridden		Eyelid ptosis	Ptosis and mydriasis
1.8	F	1	Lower limbs reduced reflexes			
2.5	F	2	Unable to run			
8	M	4	Bedridden			
5	F	2	Reduced activity and play			Sphincter incontinence
1	M	5	Requiring assisted ventilation for part of the day	Jugulum, subdiaphragmatic epigastrium tirage		
13	F	2	Reduced activity and play			
10	F	2	Reduced activity and play			
6	M	3	Able to walk 10 m		Inability to frown	
10	M	4	Chairbound			
7	M	5	Requiring assisted ventilation	Respiratory failure	Nasal voice and dysphagia.	
4	M	4	Chairbound			

**Table 4:**
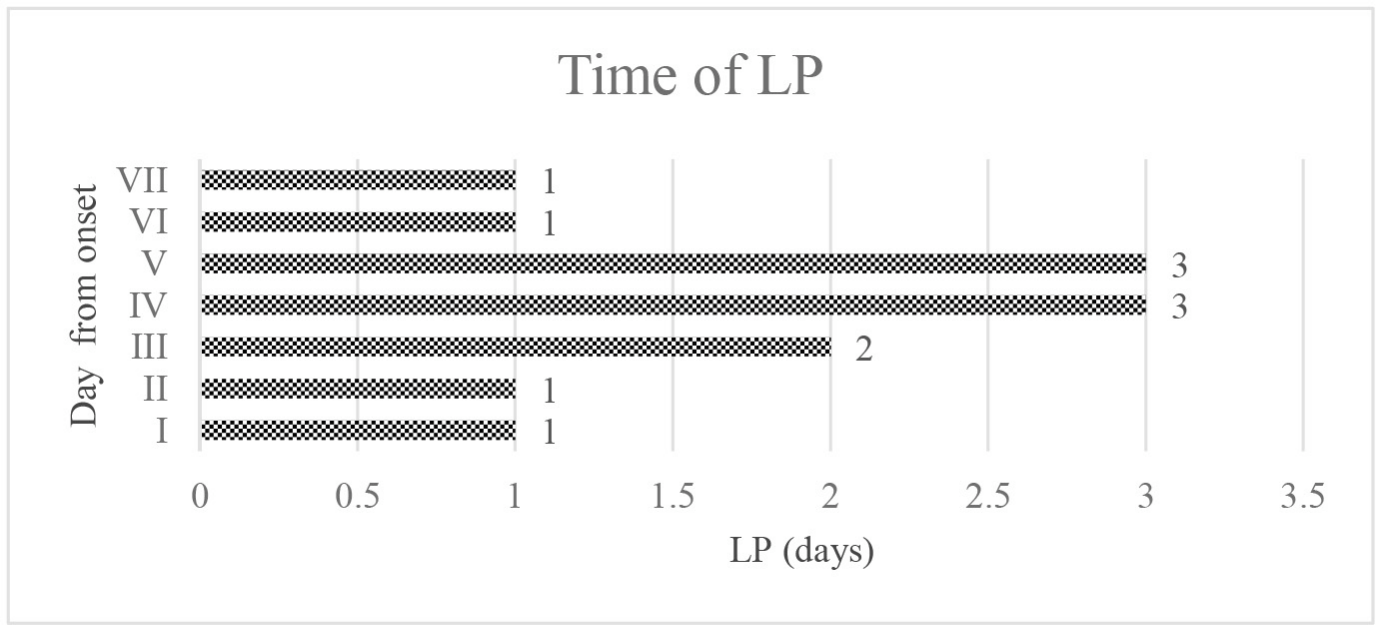
Time (days).

Lumbar puncture (LP) performed 3 days after symptoms onset.

Patients selected during this period were treated according to guidelines with standard IVIg administration of 1 g/kg daily for 2 days. In addition, patients with clinical follow-up at 2 weeks were included in the study. Data were collected and a statistical analysis was done to determine the presence of a possible correlation among these three variables.

### Statistical analysis

We performed a statistical analysis to measure the potential relationship between the clinical progress of children with GBS, MRI imaging, and CSF protein levels. We measured the correlation index between the clinical and assessed and the GBS and MRI scores according to Yakilmaz Classification. We observed that the CSF parameters that correlated with GBS disability score at 2 weeks were protein levels in the CSF, when these were increased 1, 2, and 3 times the cut-off value of 45 mg/dl as Kerasnoudis et al. reported (16).

## Results

We performed an extensive literature search and found no publications evaluating a possible correlation among the clinical severity of GBS, imaging, and laboratory values of CSF proteins combined in the same population.

Two children showed notable signs of respiratory impairment and were intubated. Three children showed involvement of the cranial nerves. Children with respiratory disturbances involving the cranial nerves had a worse clinical course (Table 1). Trigger events were represented by gastrointestinal infections, high airway infections, and vaccination of 3, 8, and 1 cases, respectively (Table 5).

**Table 5:** Clinical presentation.

**Variable**	**Patients (*n*)**
Male/Female	7/5
Age (years)	6,8
Trigger events	
-Gastrointestinal infections	3
-Airway infections	8
-Vaccination	1
Time from onset to admission (day)	4
Sensory disturbance	2
Hyporeflexia or areflexia	10

We executed the statistical analysis to measure the potential relationship among the clinical assessment of children with GBS, MRI imaging, and CSF protein levels.

It seems that these parameters have a parallel trend and a good correlation in each patient. The values of *r* calculated for all correlations are close to unity and therefore indicate a strong correlation value between them. We developed a graph to see the trend of the disability values, proteins in the CSF, and damage assessed with MRI (**Figure 4**).

### Correlation between MRI lesions and grade of disability

**Figure 3:**
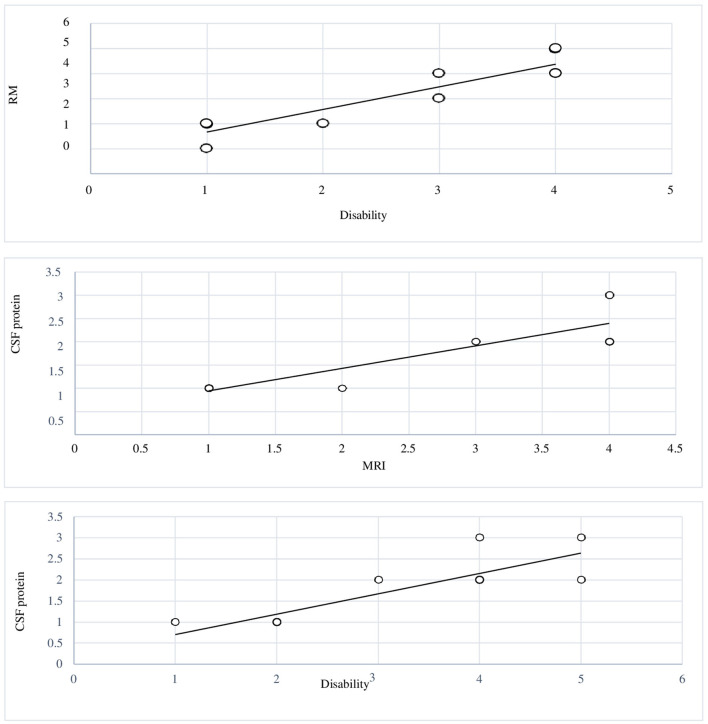
Graphic 1 showing the correlation between MRI brain lesions and levels of disability: Pearson's correlation index coefficient was 0.92. Graphic 2 The graphic showing the correlation between levels of disability and elevated CSF protein; r-value 0.85. Graphic 3 reports the correlation between MRI brain lesions and elevated CSF protein; r-value 0.87.

The graph (Figure 3) shows the correlation between brain MRI lesions according to Yikilmaz et al. classification (11) and grade of disability. Pearson's correlation index coefficient was 0.92 (*R*^2^ = 0.8573).

### Correlation between increased CSF protein and grade of disability

We assessed the chemical-physical characteristics of the CSF in each patient: appearance, color, the number of cells, glucose, proteins, presence of immunoglobulins, and albumin.

We examined these parameters one by one and the correlation with the GBS disability score and the prognosis.

The single parameter showing the more robust correlation with prognosis was the increase of the proteins in the CSF.

The graph (Figure 3) shows the correlation between levels of disability and increased levels of CSF protein. Pearson's correlation index coefficient was 0.85 (*R*^2^ = 0.7342).

### Correlations between MRI brain lesions and increased CSF protein

Our pediatric neuroradiologists team evaluated and classified magnetic resonance images following the Yakilmaz radiological classification (14). This classification presented in the study was made for descriptive purposes and the potential prognostic use was not investigated.

The graph (Figure 3) reports the correlation between MRI brain lesions and increased levels of CSF protein. The correlation index coefficient had a value of 0.87.

### Correlation among the three parameters observed in this study

**Figure 4:**
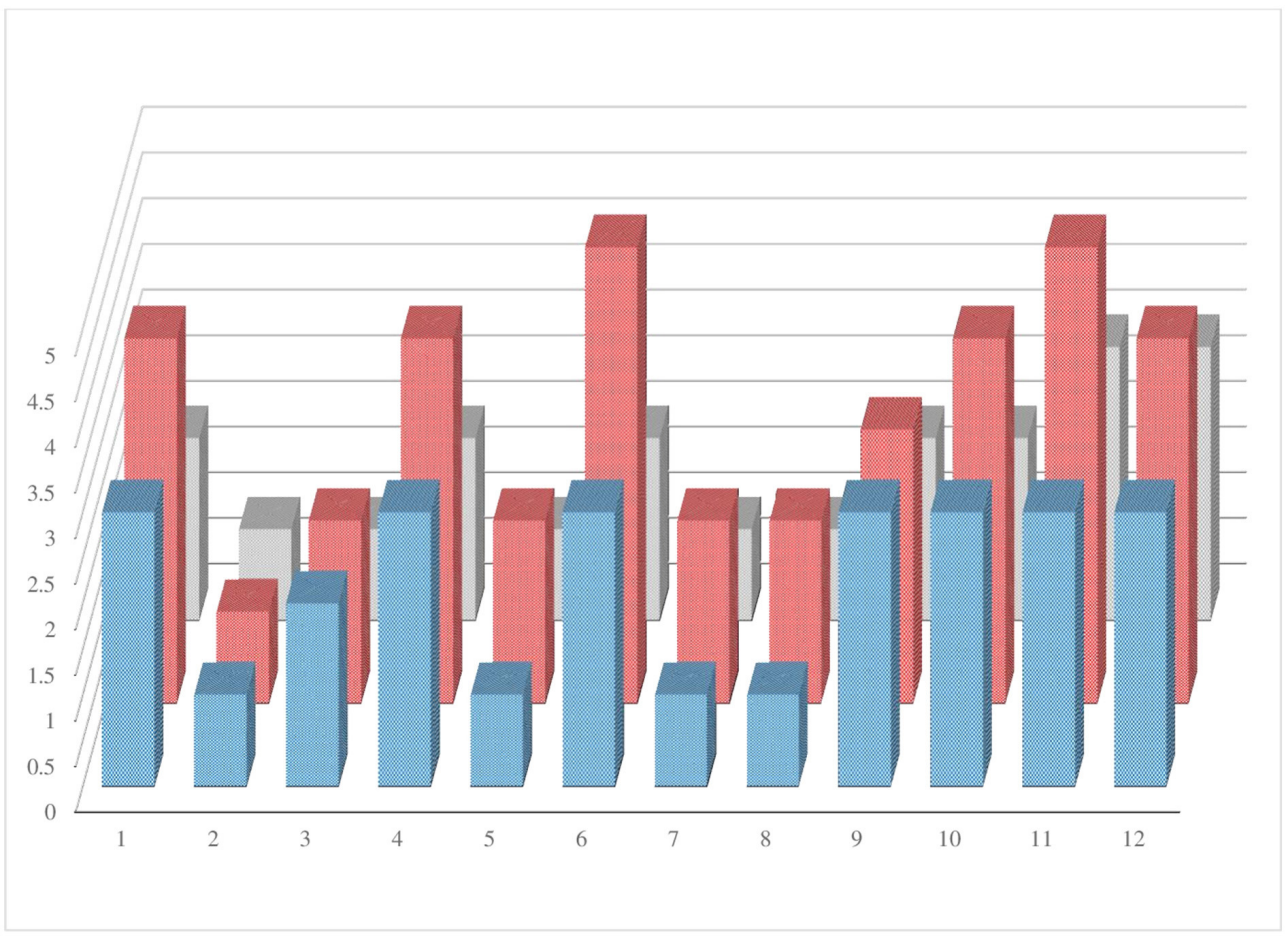
The graphic shows the trend of the three parameters studied (level of disability, MRI brain lesions, and elevated CSF protein) in each patient enrolled.

The graph (Figure 4) was performed to evaluate the trend of the three parameters studied (level of disability, MRI brain lesions, and increased levels of CSF protein) in each of the 12 patients. In the graph, the variables considered have a parallel trend in the population studied.

## Conclusion

Staging the pediatric patient affected by GBS assumes an important value both in the general evaluation of the patient and in the prognosis. Currently, prognostic scores are based exclusively on clinical parameters, such as the GBS disability scale and the Erasmus GBS scale. There is no clinical-prognostic score that takes into consideration radiological or laboratory parameters.

To fill this gap, we tried to evaluate the correlation between the imaging staging of GBS on MRI and the chemical-physical examination of the CSF of 12 pediatric patients treated.

In our evaluations, we were able to evaluate a good correlation between them.

We assessed that MRI should be considered an essential and effective exam in the diagnostic and prognostic evaluation process in GBS. The involvement of the anterior and posterior nerve root is associated with severe clinical conditions and, consequently, with a worse prognosis (17).

Brain MRI then should be performed routinely in children with GBS to estimate the possible evolution of the clinical condition.

Regarding the CFS analysis, we observed that among the various elements of cerebral liquor, the best indication for clinical condition and prognostic evaluation were related mainly to the high level of CFS protein, it has also been reported in the literature (18, 19).

The MRI and CSF laboratory parameters can be important tools for the clinician not only for diagnosis but also for having an estimate of the possible worsening of general conditions or the need to prepare measures to support life parameters.

We believe it is important to establish how early the need for ventilatory support in the most severe patient is or to be able to decide to start physiotherapy in less severe cases. The need for this study also arises to give indications to the clinician in the sub-acute phase of the disease.

Our study aimed to predict outcomes in GBS by the use of acute phase clinical features, laboratory analysis, and imaging. We have not found in the literature other studies that use laboratory, imaging, and clinical parameters to stage GBS together.

On the other hand, our systematic search of the literature did not reveal studies that simultaneously evaluate the laboratory aspects of CSF and imaging and their relationship to the clinical conditions of children with GBS.

Especially considering the ongoing International GBS Outcome Study, the potential association among CSF protein, imaging, and disability score may be worthy of further exploration (20).

The study has several limitations in our opinion: the population has a heterogeneous age and this could vary the response to the disease of different patients. In the literature, the early age of onset is a negative prognostic factor (11). The sample is numerically small, and a larger sample of patients coming from different centers would be needed for the next studies.

## Data availability statement

The raw data supporting the conclusions of this article will be made available by the authors, without undue reservation.

## Ethics statement

The study was conducted ethically in accordance with the World Medical Association Declaration of Helsinki and approved by the Ethics Committee of the University of Catania, Italy (Ethical Committee Catania 1 Clinical Registration No. 95/2018/PO). Written informed consent was obtained from the parents.

## Author contributions

PP, FP, AF, and MR worked with and helped gather patient data. FG, TT, RR, AG, GC, and ADN drafted the present manuscript. AB, GB, and ADM were the radiologist consultant. PP, ET, MS, and AG were responsible for revising the work critically for important intellectual content. All authors read and approved the final manuscript.

## Conflict of interest

The authors declare that the research was conducted in the absence of any commercial or financial relationships that could be construed as a potential conflict of interest.

## Publisher's note

All claims expressed in this article are solely those of the authors and do not necessarily represent those of their affiliated organizations, or those of the publisher, the editors and the reviewers. Any product that may be evaluated in this article, or claim that may be made by its manufacturer, is not guaranteed or endorsed by the publisher.
